# Variations in Soil Microbial Biomass Carbon and Soil Dissolved Organic Carbon in the Re-Vegetation of Hilly Slopes with Purple Soil

**DOI:** 10.1371/journal.pone.0166536

**Published:** 2016-12-15

**Authors:** Ning Yang, Dongsheng Zou, Manyuan Yang, Zhonggui Lin

**Affiliations:** 1 College of Landscape Architecture, Hunan Environmental-Biological Polytechnic College, Hengyang, China; 2 College of Bioscience and Biotechnology, Hunan Agricultural University, Changsha, China; University of Vigo, SPAIN

## Abstract

Crust restoration is increasingly being done but we lack quantitative information on soil improvements. The study aimed to elucidate the dynamics involving soil microbial biomass carbon and soil dissolved organic carbon in the re-vegetation chronosequences of a hillslope land with purple soil in Hengyang, Hunan Province. The soil can cause serious disasters with both soil erosion and seasonal drought, and also becomes a typical representative of ecological disaster area in South China. Using the space-for-time method, we selected six typical sampling plots, designated as follows: grassplot community, meadow thicket community, frutex community, frutex and arbor community, arbor community, and top-level vegetation community. These plots were established to analyze the changes in soil microbial biomass carbon, soil microbial quotien, dissolved organic carbon, dissolved organic carbon/soil organic carbon, and soil basal respiration in 0–10, 10–20, and 20–40 cm soil layers. The relationships of these parameters with soils physic-chemical properties were also determined. The ecological environment of the 6 plant communities is similar and typical; they denoted six different successive stages of restoration on hillslopes with purple soils in Hengyang, Hunan Province. The soil microbial biomass carbon and soil basal respiration contents decreased with increasing soil depth but increased with re-vegetation. By contrast, soil microbial quotient increased with increasing soil depth and re-vegetation. From 0–10 cm soil layer to 20–40 cm soil layer, the dissolved organic carbon content decreased in different re-vegetation stages. In the process of re-vegetation, the dissolved organic carbon content increased in the 0–10 and 10–20 cm soil layers, whereas the dissolved organic carbon content decreased after an initial increase in the 20–40 cm soil layers. Meanwhile, dissolved organic carbon/soil organic carbon increased with increasing soil depth but decreased with re-vegetation. Significant correlations existed among soil microbial biomass carbon, soil microbial quotient, dissolved organic carbon, soil basal respiration and soil physic-chemical properties associated with soil fertility. The results showed that re-vegetation was conducive to the soil quality improvement and the accumulation of soil organic carbon pool of the hillslope land with purple soil in Hengyang, Hunan Province.

## Introduction

Soil organic carbon is regarded as the material basis of soil fertility, and it has also been the key content of investigations on the global carbon cycle and climate change. Soil microbial biomass carbon and soil dissolved organic carbon are usually used to characterize the activity of soil organic carbon, so they are given considerable attention in different studies [1, 2]. In terms of the energy cycle and nutrient transfer, soil microbial biomass carbon is extremely sensitive and reflects small alterations in soil organic matter earlier than the total carbon changes [3, 4]. Dissolved organic carbon is a sensitive indicator of the changes in soil environment. It also provides the kind of energy that can be utilized directly by microorganisms. Moreover, dissolved organic carbon influences the transformation and degradation of organic and inorganic substances in soils [5–7]. Therefore, the study of soil microbial biomass carbon and dissolved organic carbon is of great significance for vegetation restoration.

The hillslope landwithpurplesoilcovers1.625 × 10^3^km^2^, accounting for 25% of the land area in Hengyang of Hunan Province, China. The parent rocks of purple soil including the purple shale, purple sandstone and purple sandy conglomerate are mostly continental or lacustrine deposits in the Cretaceous and Third periods. The formation of the rock strata is mostly based on the combination of shale, sandstone and mudstone with mutual layered arrangement. This lithology combination leads to the great differences in expansion coefficient. Frequent expansion and shrinkage will happen in high temperature, wet and dry climate alternation conditions, easily causing physical disintegration. Consequently, the rocks will be peeled into debris from the outside to the inside, and purple soil is formed after further weathering. The purple soil is vulnerable to erosion. It has short development period, and poor soil fertility as the content of organic matter and nitrogen content is low. Also, it is often in its infancy with deep color and strong heat absorption. In summer, it has high ground temperature and big evaporation. All these in addition to other adverse environmental impacts such as regional water and heat distributions plus unreasonable development result in not only the sparse vegetation for a long time (there is even a large area of exposed bedrock in some regions with almost no soil development layer and extremely difficult vegetation restoration) but also severe soil erosion and seasonal drought in hillslope land with purple soil. The land has become the serious disaster area with both soil erosion and seasonal drought in Hunan province, China, and also a typical representative of ecological disaster area in South China [8]. Severe ecological environment restricts the development of local rural economy seriously since the income of farmers is significantly lower than that in other areas, and even endangers the survival of local farmers. In order to improve the ecological environment of the hilly slopes with purple soil, regional governments at all levels have spent a total of nearly 1.5 billion yuan, taking the measures of returning farmland to forest, and afforestation through digging trenches in the mountains, and picking and filling external soil since the 90s of the last century. However, because tree species and soil were not compatible and artificial forest stand structure is simple, most of the results are minimal, resulting in most of hilly slopes with purple soil still in barren state. Re-vegetation restoration is the key measure to control the ecological environment of the region. Currently, a large number of studies have investigated the physical and chemical properties of soil surfaces [9], soil enzyme activity [10], and quantity of soil microorganisms [11] during the vegetation restoration of the hilly slopes in the purple soil areas. However, the soil microbial biomass carbon and dissolved organic carbon characteristics and the relationship with other soil properties during the vegetation restoration of the hilly slopes in the purple soil areas are rarely reported. The method of “spatial sequence instead of time series” [12] was adopted in this study to explore the variations in soil microbial biomass carbon and dissolved organic carbon of 0–10, 10–20 and 20–40 cm soil layers in the different stages of vegetation restoration of the hilly slopes in the purple soil area of Hengyang, Hunan Province. The findings would provide a scientific basis for vegetation restoration.

## Study Area

**Fig 1:**
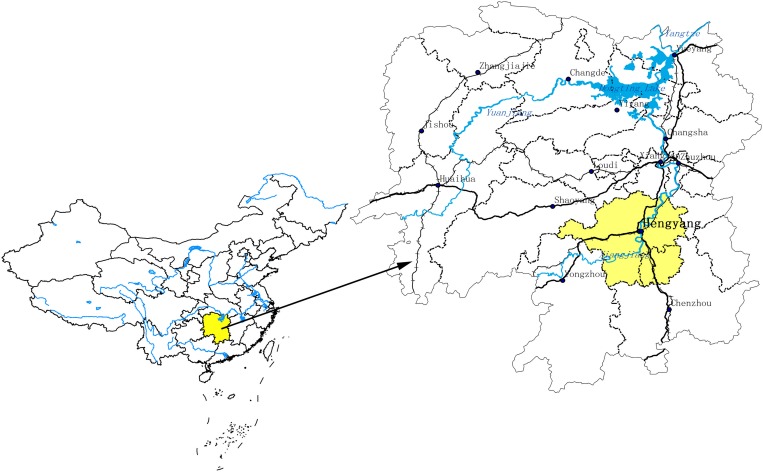
The schematic chart of the study area.

The hilly slope covered with purple soil in Hengyang (Fig 1) is located in the southern and central parts of Hunan Province and in the middle reaches of Xiangjiang River, with geographic coordinates ranging from 110°32′16″E to 113°16′32″E and 26°07′05″N to 27°28′24″N. This study area was approved by the Forestry Bureau of Hengyang, Hunan Province of China. The purple soil is distributed in a reticular formation in the middle of the abovementioned region, with altitudes ranging from 60 m to 200 m. The area experiences a subtropical monsoon climate, with an annual average temperature of 18°C. The maximum temperature is 40.5°C, and the minimum temperature is −7.9°C. The annual average rainfall is 1325 mm, whereas the annual average evaporation is 1426.5 mm. The average relative humidity is 80%, whereas the annual frost-free period is 286 days.

## Methods

### Selection of sample plot

The natural restoration process of the degraded community in this area is characterized by six stages, namely, the grassplot community, meadow thicket community, frutex community, frutex and arbor community, arbor community, and top-level vegetation community [10, 11]. Based on local records, the lower parts of the slopes with basically identical ecological factors, such as slope gradient, slope direction, slope position, and bare rock ratio, were selected. Representative sample plots were set along the contour to represent the different stages of restoration. The area of each sample plot is greater than 1 hm^2^, and the sample plots are all abandoned lands in early succession. The basic information of the plant community at each stage is shown in Table 1. Typical sample plots were set up with an area of 900m^2^ (30m×30m) in each re-vegetation stage. Altogether, 18 sample plots were established for three experimental replicates.

**Table 1:** The basic information of sample plots.

Re-vegetation stages	Grassplot community	Meadow thicket community	Frutex community	Frutex and arbor community	Arbor community	Top-level vegetation community
**Geographic coordinates**	111°32′16″E, 26°57′43″N	112°47′32″E, 27°27′05″N	113°12′2″E, 27°8′54″N	112°5′43″E, 26°57′15″N	110°58′45″E, 26°45′26″N	112°43′16″E, 27°8′47″N
**Slope(°)/Aspcet**	25–35/SW	35/SW	20–30/SW	25/SW	25–35/SW	20–30/SW
**Altitude(m)**	155	125	140	145	110	100
**Coverage(%)**	≈25	45	≈55	≈70	≈80	>85
**Dominant plant**	Only an herb layer mainly composed of *Setaria viridis*, *Miscanthus sinensis*, and *Prunella vulgaris*.	The community is composed of herbs and shrubs. The herbs mainly comprise *S*. *viridis* and *P*. *vulgaris*, whereas the dominant shrubs include *Lagerstroemia indica*, *Abelia chinensis*, *Pyracantha fortuneana*, and *Coriaria nepalensis*.	The plant community is a shrub layer mainly composed of *Vitex negundo* var. ‘cannabifolia’, *L*. *indica*, *Ligustrum luciduum*, *Serissa foetida*, and *C*. *nepalensis*.	The stand level exhibits obvious structural differentiation. The shrub layer chiefly consists of *V*. *negundo* var. *cannabifolia*, *Sapium rotundifolium*, *Populus davidiana*, *Ardisia japonica*, and *C*. *nepalensis*. Moreover, rattan spines are mainly found on the surface.	The tree layer primarily comprises *Liquidambar formosana*, *S*. *rotundifolium*, *Bischofia polycarpa*, *P*. *davidiana*, and *Quercus* spp. The shrub layer is mainly composed of *V*. *negundo* var. *cannabifolia*, *Serissa foetida*, and *Dendranthema* spp.	The dominant species is *L*. *formosana*.
**Re-vegetation years(a)**	2	5–8	15–20	25–30	35–40	≈50

### Soil sample collection and processing

In the middle of September in 2014, the “S”-type five-point hybrid sampling method was adopted for soil sampling along two diagonals of 18 sample plots. Samples were obtained from three layers, namely, the upper (0–10 cm), middle (10–20 cm), and lower layers (20-40cm). Soil samples from the same layer were mixed, giving a total of 108 mixed samples (2 diagonals × 3 soil layers × 18 sample plots). When sampling was performed, surface litter, visible stones, and plant roots were removed. Some of the soil samples were used to fill up aluminum boxes for the determination of the soil water content, and the rest were brought back to the lab in sealed plastic bags. Among these samples, a portion was air dried for soil organic carbon determination, whereas another portion was stored in the refrigerator at 4°C for the determination of soil basal respiration, soil microbial carbon, and dissolved organic carbon.

### Research methods

The determination of soil physical and chemical properties involved the following measurements [13]. Soil water content was determined using the drying method (at 105°C for 12 h). Soil bulk density was obtained using the cutting ring method, whereas soil organic carbon was measured through potassium dichromate oxidation and heating [14]. Total nitrogen was measured using the semi-micro Kjeldahl method [14], whereas total phosphorus was determined via NaOH fusion, molybdenum–antimony coloring, and UV spectrophotometry [14]. Total potassium was ascertained through NaOH fusion and atomic absorption [14], and pH was measured using electrodepotential analysis. The physical and chemical properties of the soils in the different stages of restoration are shown in Table 2. The soil microbial properties were determined using the following measurements. Soil microbial biomass carbon was determined through chloroform fumigation and K_2_SO4 extraction (conversion coefficient K is 0.45) [15]. Culturing in closed containers and alkali absorption were employed to obtain soil basal respiration [16]. The soil microbial quotient was calculated from the ratio of soil microbial biomass carbon to soil organic carbon (Cmic/Corg). Dissolved organic carbon was measured through colorimetric analysis. In this process, fresh soil samples, with a soil to water ratio of 5:1, were prepared and oscillated for 2 h in a 200 rpm oscillator. The samples were centrifuged for 20 min at 12,500 rpm and filtered through a 0.45 μm filtration film. The dissolved organic carbon of the filtrate was then determined [17].

**Table 2:** Soil physicochemical properties in different re-vegetation stages.

Soil property	Soil layer (cm)	Re-vegetation stages					
		Grassplot community	Meadow thicket community	Frutex community	Frutex and arbor community	Arbor community	Top-level vegetation community
**SWC (g∙kg**^**−1**^**)**	0–10	134.9Fa	145.6Ea	177.9Da	200.7Ca	232.0Ba	287.5Aa
	10–20	123.7Fab	135.2Eab	162.9Dab	180.4Cb	219.4Bb	264.7Ab
	20–40	110.4Fb	128.0Eb	154.0Db	175.3Cc	200.0Bc	243.7Ac
**SBD (g∙cm**^**−3**^**)**	0–10	1.432Ab	1.400Bc	1.398Bb	1.207Cb	1.187Db	1.100Eb
	10–20	1.501Ab	1.431Bb	1.419Cab	1.400Ca	1.210Dab	1.105Eab
	20–40	1.502Aa	1.439Ba	1.412Ca	1.405Ca	1.298Da	1.108Ea
**SOC (mg∙kg**^**−1**^**)**	0–10	32.12Ea	26.58Fa	33.38Da	49.23Ba	59.82Aa	48.96Ca
	10–20	19.64Eb	16.93Fb	21.70Db	29.39Cb	32.26Ab	30.56Bb
	20–40	14.15Dc	13.46Ec	14.74Dc	19.21Bc	25.78Ac	18.79Cc
**TN (%)**	0–10	0.66Da	0.67Da	0.75Ca	0.89Ba	1.05Ba	1.29Aa
	10–20	0.54Fb	0.59Eb	0.74Da	0.79Cb	1.04Bb	1.28Aa
	20–40	0.31Fc	0.48Ec	0.69Db	0.77Cb	1.04Bb	1.28Aa
**TP (%)**	0–10	0.07Ca	0.07Ca	0.08Ba	0.07Ca	0.09Aa	0.09Aa
	10–20	0.07Ba	0.06Cab	0.08Aa	0.06Cb	0.08Ab	0.08Ab
	20–40	0.05Db	0.05Db	0.07Bb	0.06Cb	0.07Bc	0.08Ab
**TK (%)**	0–10	2.11Aa	2.09Aa	2.12Aa	2.13Aa	2.10Aa	2.13Aa
	10–20	2.12Aa	2.10Aa	2.09Aa	2.13Aa	2.11Aa	2.12Aa
	20–40	2.10Aa	2.09Aa	2.11Aa	2.09Aa	2.09Aa	2.10Aa
**pH**	0–10	8.21Aa	8.15Aa	8.09ABb	8.00ABb	7.54Ba	7.12Ba
	10–20	8.13Ab	8.12Aa	8.10Aab	8.06Bab	7.52Ca	7.13Ca
	20–40	8.13Ab	8.12Aa	8.12Aa	8.11Aa	7.55Ba	7.13Ba

Note: Different capital letters on the same row indicate a significant difference among different re-vegetation stages, and different small letters denote a significant difference among the different soil layers at 0.05.

### Data processing

SPSS13.0 was used for statistical analysis. One-way ANOVA and least significant difference were adopted to compare the differences between different data (α = 0.05).Pearson correlation coefficient was employed to analyze the correlation coefficients of different factors. All of the data in the tables were expressed as the average of three repetitions±standard deviation.

## Results

### Characteristics of soil microbial biomass carbon in vegetation restoration

#### Soil microbial biomass carbon content

Table 3 shows that the soil microbial biomass carbon levels of the different vegetation restoration stages in the three soil layers (0–10, 10–20, and 20–40 cm) were arranged in descending order as follows: top-level vegetation community, arbor community, frutex and arbor community, frutex community, meadow thicket community, and grassplot community (*P*<0.05). This result indicated that soil microbial biomass carbon increased with the progression of vegetation restoration. Moreover, the soil microbial biomass carbon content decreased with soil depth (Table 3), thereby implying the obvious surface accumulation of the soil microbial biomass carbon. The ratios of the soil microbial biomass carbon of the 20–40 cm soil layer to that of the 0–10cm soil layer from grassplot community to top-level vegetation community were 0.51, 0.51, 0.75, 0.74, 0.79, and 0.97, respectively. Meanwhile, the ratios of the soil microbial biomass carbon of the 10–20cm soil layer to that of the 0–10cm soil layer from grassplot community to top-level vegetation community were 0.66, 0.67, 0.81, 0.76, 0.90, and 0.90, respectively. As restoration progressed, the proportions of soil microbial biomass carbon in the soil layers increased with depth, and the soil microbial biomass carbon in the different soil layers began to show homogenization.

**Table 3:** Soil microbial biomass carbon contents in different re-vegetation stages (mg∙kg^−1^).

Soil layer (cm)	Re-vegetation stages					
	Grassplot community	Meadow thicket community	Frutex community	Frutex and arbor community	Arbor community	Top-level vegetation community
**0–10**	389.09±30.12Fa	390.10±21.09Ea	798.54±49.25Da	1100.32±65.63Ca	1224.36±32.12Ba	1334.76±32.12Aa
**10–20**	256.87±21.87Eb	260.64±17.37Eb	650.36±42.98Db	831.64±45.17Cb	1186.46±87.34Bb	1221.09±54.09Ab
**20–40**	198.27±12.00Ec	200.42±21.00Ec	597.34±45.82Dc	812.69±56.00Cb	1046.12±98.07Bc	1200.34±98.56Ab

Note: Different capital letters on the same row indicate a significant difference among different re-vegetation stages, and different small letters denote a significant difference among the different soil layers at 0.05.

#### Soil microbial quotient

It can be seen from Table 4, the variations of soil microbial quotient in each soil layers are similar to those of soil microbial biomass carbon. The order of soil microbial quotient form large to small in different re-vegetation stages was top-level vegetation community>frutex community>frutex and arbor community>arbor community>meadow thicket community>grassplot community (*P*< 0.05) at 0–10 cm soil layer, top-level vegetation community>arbor community>frutex community>frutex and arbor community>meadow thicket community>grassplot community (*P*<0.05) at 10–20 cm soil layer, and top-level vegetation community>frutex and arbor community>frutex community (≈arbor community) >meadow thicket community>grassplot community (*P*< 0.05) at 20–40 cm soil layer. Those showed soil microbial quotient increases with the re-vegetation. The order of soil microbial quotient at soil layers was that at 20–40 cm soil layer >that at 10–20 cm soil layer >that at 0–10 cm soil layer (*P*< 0.05), demonstrating the increase in soil microbial quotient with soil depth. The results showed that the soil microbial activity would become increasingly strong with the re-vegetation. Particularly, the increase on soil microbial activity rate at 20–40 cm soil layer is very significant.

**Table 4:** Soil microbial quotient in different re-vegetation stages.

Soil layer (cm)	Re-vegetation stages					
	Grassplot community	Meadow thicket community	Frutex community	Frutex and arbor community	Arbor community	Top-level vegetation community
**0–10**	12.12±1.09Fc	14.68±1.33Eb	23.93±2.00Bc	22.35±2.09Cc	20.47±2.31Dc	27.26±2.54Ac
**10–20**	13.08±1.00Fb	14.89±1.09Eb	29.97±2.18Cb	28.30±1.87Db	36.78±3.54Bb	39.96±4.00Ab
**20–40**	14.01±1.23Ea	15.39±0.95Da	40.53±3.98Ca	42.31±3.99Ba	40.58±4.12Ca	63.90±5.12Aa

Note: Different capital letters on the same row indicate a significant difference among different re-vegetation stages, and different small letters denote a significant difference among the different soil layers at 0.05.

### Characteristics of dissolved organic carbon in vegetation restoration

#### Dissolved organic carbon content

From Table 5, it can be seen that the order of dissolved organic carbon in different re-vegetation stages was top-level vegetation community (≈frutex community, frutex and arbor community, arbor community)>meadow thicket community (≈grassplot community) (*P*< 0.05) at 0–10 cm and 10–20 cm soil layers, and frutex community (≈frutex and arbor community)>grassplot community(≈meadow thicket community, top-level vegetation community)>arbor community (*P*< 0.05) at 20–40 cm soil layer. The order of dissolved organic carbon at different soil layers was that at 0–10 cm soil layer >that at 10–20 cm soil layer >that at 20–40 cm soil layer (*P*< 0.05) in the grassplot community, meadow thicket community, frutex community, and frutex and arbor community Besides, dissolved organic carbon would significantly decrease with the increase in soil depth (*P*< 0.05). The results showed that dissolved organic carbon would increases with the re-vegetation at 0–10 cm and 10–20 cm soil layers, and firstly increase, then decrease, and again increase with the re-vegetation at 20–40 cm soil layer. In addition, dissolved organic carbon would decrease with the increase in soil depth in all the re-vegetation stages.

**Table 5:** Dissolved organic carbon contents in different re-vegetation stages (mg∙kg^−1^).

Soil layer (cm)	Re-vegetation stages					
	Grassplot community	Meadow thicket community	Frutex community	Frutex and arbor community	Arbor community	Top-level vegetation community
**0–10**	58.89±5.23Ba	58.96±5.12Ba	60.41±5.21Aa	60.55±4.28Aa	60.42±5.00Aa	60.71±4.01Aa
**10–20**	58.74±4.09Ba	58.75±5.30Ba	60.32±4.06Aa	60.25±6.12Aa	60.00±3.09Aa	59.97±4.37Aab
**20–40**	58.30±4.07Ba	58.69±4.99Ba	60.13±6.00Aa	60.12±5.32Aa	55.43±3.98Cb	58.61±3.88Bb

Note: Different capital letters on the same row indicate a significant difference among different re-vegetation stages, and different small letters denote a significant difference among the different soil layers at 0.05.

#### Proportion of dissolved organic carbon in soil organic carbon

As shown in Table 6, the order of dissolved organic carbon/soil organic carbon in different re-vegetation stages was meadow thicket community>grassplot community (≈frutex community)>frutex and arbor community (≈top-level vegetation community)>arbor community (*P*<0.05) at 0–10 cm soil layer, meadow thicket community>grassplot community>frutex community>frutex and arbor community>top-level vegetation community>arbor community (*P*<0.05) at 10–20 cm soil layer, and meadow thicket community>grassplot community (≈frutex community)>frutex and arbor community (≈top-level vegetation community)>arbor community (*P*<0.05) at 20–40 cm soil layer. The order of dissolved organic carbon/soil organic carbon at soil layers was that at 20–40 cm soil layer >that at 10–20 cm soil layer >that at 0–10 cm soil layer (*P*< 0.05) in all re-vegetation stages. The results showed that dissolved organic carbon/soil organic carbon would decrease with the re-vegetation, and increase with the increase in soil depth.

**Table 6:** Dissolved organic carbon/soil organic carbon in different re-vegetation stages.

Soil layer (cm)	Re-vegetation stages					
	Grassplot community	Meadow thicket community	Frutex community	Frutex and arbor community	Arbor community	Top-level vegetation community
**0–10**	1.81±0.14Bc	2.21±0.54Ac	1.81±0.15Bc	1.23±0.11Cc	1.01±0.17Dc	1.24±0.13Cc
**10–20**	2.98±0.20Bb	3.47±0.39Ab	2.78±0.23Cb	2.05±0.12Db	1.86±0.16Fb	1.96±0.17Eb
**20–40**	4.12±0.44Ba	4.36±0.41Aa	4.08±0.47Ba	3.13±0.43Ca	2.15±0.15Da	3.12±0.32Ca

Note: Different capital letters on the same row indicate a significant difference among different re-vegetation stages, and different small letters denote a significant difference among the different soil layers at 0.05.

### Soil basal respiration in vegetation restoration

From Table 7, it can be seen that the order of soil basal respiration in different re-vegetation stages at each soil layer was top-level vegetation community>frutex and arbor community>arbor community>frutex community>meadow thicket community>grassplot community (*P*<0.05). The order of soil basal respiration at all soil layers in each re-vegetation stage was that at 0–10 cm soil layer >that at 10–20 cm soil layer >that at 20–40 cm soil layer (*P*< 0.05). The results showed that soil basal respiration would become stronger with the re-vegetation, and weaker with the increase in soil depth [18].

**Table 7:** Soil basal respiration in different re-vegetation stages (mg∙kg^−1^∙h^−1^).

Soil layer (cm)	Re-vegetation stages					
	Grassplot community	Meadow thicket community	Frutex community	Frutex and arbor community	Arbor community	Top-level vegetation community
**0–10**	1.12±0.12Fa	1.26±0.11Ea	1.54±0.09Da	2.04±0.11Ba	1.69±0.12Ca	2.48±0.19Aa
**10–20**	0.65±0.09Fab	0.71±0.18Eb	0.87±0.08Db	1.63±0.09Bb	1.43±0.13Cb	1.71±0.18Ab
**>20**	0.23±0.08Fb	0.37±0.02Ec	0.64±0.08Dc	1.40±0.07Bc	1.09±0.09Cc	1.58±0.18Ac

Note: Different capital letters on the same row indicate a significant difference among different re-vegetation stages, and different small letters denote a significant difference among the different soil layers at 0.05.

### Analysis of the correlation between the soil microbial properties and the physical and chemical properties

As shown in Table 8, Soil bulk density has significant negative correlationships (*P*< 0.05 or *P*< 0.01) with soil microbial biomass carbon, soil microbial quotient, dissolved organic carbon, and soil basal respiration. soil microbial biomass carbon had significant positive correlationships (*P*< 0.01) with soil water content, soil organic carbon, total nitrogen and total phosphorus, while both soil microbial quotient and soil basal respiration had significantly positive correlationships (*P*< 0.05) with soil water content, soil organic carbon, total nitrogen and total phosphorus. These indicated that with the progress of re-vegetation, the soil quality was considerably improved, and soil organic carbon changed in the direction of large quantity and good quality with the benign development of the ecological environment of the soil. Also, the change of soil microbial biomass carbon was greater than that of dissolved organic carbon.

**Table 8:** Correlation coefficients between soil physico-chemical properties and soil microbial characteristics.

Soil microbial characteristics	Soil physicochemical properties						
	Soil water content	Soil bulk density	Soil organic carbon	Total nitrogen	Total phosphorus	Total potassium	pH
**Soil microbial biomass carbon**	0.900**	−0.887**	0.901**	0.900**	0.944**	0.449	−0.238
**Soil microbial quotient**	0.612*	−0.559*	0.610*	0.599*	0.606*	0.318	−0.403
**Dissolved organic carbon**	0.687*	−0.643*	0.679*	0.670*	0.598*	0.337	−0.309
**Soil basal respiration**	0.554*	−0.600*	0.600*	0.587*	0.599*	0.389	−0.248

Note

**P < 0.01

* *P* < 0.05.

## Discussion and Conclusion

### Variations in the soil microbial biomass carbon and dissolved organic carbon contents with the progression of vegetation restoration and their significance

This study demonstrated that soil microbial biomass carbon increased with restoration and decreased with the increase in soil depth. This finding was consistent with the results of a study by Shao*et al*. [19] on *Pinus tabulaeformis* planted in the Huangfuchuan Watershed;a study by Li*et al*. [20] on planted and Chinese fir forests in Sanming Kawakamii County, Fujian Province; and a study by Liuand Wang [21] on temperate forests. soil microbial quotient, as a sensitive indicator of the quality of the soil carbon pool, could reflect more effectively the effect of re-vegetation on the behaviour of soil carbon, compared with soil microbial biomass carbon. A previous research [22] pointed that higher soil microbial quotient indicated carbon accumulation in the soil, and the microbial number and population structure in the soil changed with the re-vegetation, which would slow down the conversion of non active organic carbon to active organic carbon, and ultimately increase the soil organic carbon. Soil microbial quotient increased with the progress of the re-vegetation, indicating that the soil carbon element was effective and carbon sequestration capacity was increased, and thus soil organic carbon was gradually accumulated. Soil microbial quotient increased with the increase in soil depth, denoting that the presence of more active carbon pool in sub-surface soil. Some researchers have reported the sub-surface soil acts as store of microbial inoculation. This observation was consistent with the results of a study by Yi*et al*. [23] on the forests of the Dinghu Mountain Nature Reserve and a study by Wei*et al*. [24] on the forests of a karst area in Guizhou Province.

Our results also showed that dissolved organic carbon at 0–10 cm soil layer increased with the progress of re-vegetation, which coincided closely with the findings from Gong *et al*. [25] on a tropical broad-leaved evergreen forest in Central Asia. Moreover, dissolved organic carbon decreased with the increase in soil depth was consistent with the findings of Jiang [26] on red-yellow soil in Linglong Mountain, Lin’an City, Zhejiang Province, China. He found that the conversion of soil organic carbon to dissolved organic carbon at 10–20 cm and 20–40 cm soil layer can be effectively reduced, which has positive significance for the accumulation of soil organic carbon. dissolved organic carbon /soil organic carbon is commonly used as a good indicator of the turnover of soil bioactive organic carbon pool, and the greater the value, the easier the decomposion of soil organic matter by soil microbes. Therefore, the findings from this study of dissolved organic carbon /soil organic carbon decrease with the progress of re-vegatation and its increase with the increase on soil depth meant that the turnover rate of bioactive soil organic carbon pool had the same trends. Also, those and that the conversion rates of soil non active organic carbon to the active organic carbon indicated with soil microbial quotient with the progress of re-vegetation slowed down confirmed each other [27]. In short, the variations of soil microbial biomass carbon and dissolved organic carbon had an important significance in indicating soil organic carbon changes in the process of re-vegetation in the hilly slopes with purple soil in Hengyang City.

### Relationship of the co-evolution between soil microbial biomass carbon, dissolved organic carbon and other soil properties in vegetation restoration

According to this study, good co-evolutionary relationships between soil microbial biomass carbon, dissolved organic carbon and other physical, chemical, and biological soil characteristics were found. Among these properties, soil basal respiration reflects the biological characteristics and the metabolic intensity of substances in soil [28, 29], which can characterize the activity of soil microbial biomass carbon and dissolved organic carbon to some extent. Soil basal respiration, soil microbial biomass carbon, and dissolved organic carbon have highly significant or significant positive correlations with soil water content, soil organic carbon, total nitrogen, and total phosphorus and highly significant or significant negative correlations (Table 8) with soil bulk density. These results further confirmed the co-evolutionary relationships. This research showed that soil basal respiration increased with the succession of vegetation restoration and decreased with soil depth, indicating that soil microbial activity followed the same trend. This finding was consistent with the result of a study by Zhao *et al*. [30] on the bamboo forests of Jinyun Mountain in Chongqing Province. Meanwhile, the result that soil microbial biomass carbon and dissolved organic carbon had highly significant or significant (Table 8) relationships with soil organic carbon, total nitrogen, and total phosphorus was basically consistent with the result of a study by Liu*et al*. [31] on planted *P*.*tabulaeformis*. The present study indicated that, to some extent, soil microbial biomass carbon and dissolved organic carbon were dependent on the improvement of the soil organic carbon content and soil properties. From another perspective, a close relationship between soil microbial biomass carbon and dissolved organic carbon may exist and partially reflect the active carbon content in soil.
